# Clopidogrel vs. Aspirin in Double Antithrombotic Therapy for Patients on Oral Anticoagulation Undergoing Coronary Stenting

**DOI:** 10.3390/jcdd13060249

**Published:** 2026-06-05

**Authors:** Graziella Pompei, Manfredi Arioti, Carolina Moretti, Francesco Bendandi, Riccardo Panevino, Sebastiano Sanna, Gavino Casu, Andrea Rubboli

**Affiliations:** 1 Division of Cardiology, Department of Emergency, Internal Medicine and Cardiology, Santa Maria delle Croci Hospital, Viale Vincenzo Randi 5, 48121 Ravenna, Italy; graziella.pompei@auslromagna.it (G.P.); francesco.bendandi2@auslromagna.it (F.B.); riccardo.panevino@studio.unibo.it (R.P.); 2 Cardiovascular Institute, Azienda Ospedaliero—Universitaria di Ferrara, Via Aldo Moro 8, 44124 Ferrara, Italy; 3 Department of Medical and Surgical Sciences (DIMEC), University of Bologna, 40100 Bologna, Italy; 4 Clinical and Interventional Cardiology, Department of Medicine, Surgery and Pharmacy, University Hospital, Viale San Pietro 10, 07100 Sassari, Italy; 5 Faculty of Medicine, University of Bologna, Campus of Ravenna, 48121 Ravenna, Italy

**Keywords:** oral anticoagulant, atrial fibrillation, triple antithrombotic therapy, double antithrombotic therapy, percutaneous coronary intervention, dual antiplatelet therapy

## Abstract

Over the past two decades, the combined use of long-term anticoagulation and antiplatelet therapy following percutaneous coronary intervention has been extensively investigated. Efforts to define an optimal antithrombotic strategy—balancing protection against thrombotic and thromboembolic events with minimization of bleeding risk—have led to the design and conduct of randomized clinical trials. This narrative review synthesizes the main evidence comparing different antithrombotic approaches in this setting, with particular focus on regimens stratified by oral anticoagulant type and on the direct comparison between aspirin- and clopidogrel-based double antithrombotic therapy, as evaluated in a limited number of recent studies. Further large-scale randomized data comparing these two regimens are needed to strengthen the current evidence and clarify this issue, as well as to evaluate the role of platelet function and/or genetic testing in guiding the selection of the optimal antiplatelet agent.

## 1. Introduction

**Figure 1:**
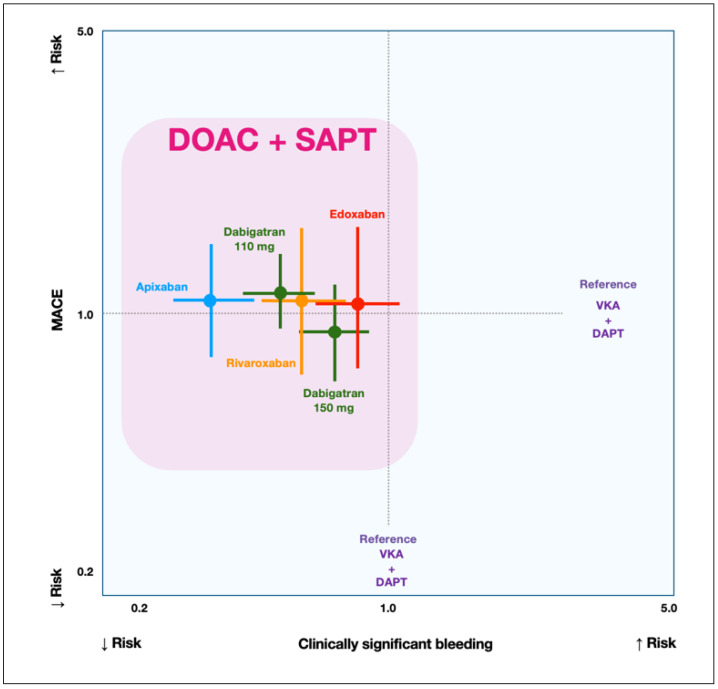
Randomized controlled trials consistently demonstrating a lower risk of clinically significant bleeding with double antithrombotic therapy (DAT) compared to triple antithrombotic therapy (TAT), yet with a trend towards an increased risk of MACEs. Adapted from Capodanno et al., J Am Heart Assoc 2020 [18]. Abbreviations. DOAC, direct oral anticoagulant; SAPT, single antiplatelet therapy; MACE, major adverse cardiovascular event; VKA, vitamin K antagonist; DAPT, dual antiplatelet therapy.

Beginning in the early 2000s, the population of patients on long-term oral anticoagulation (OAC), most commonly for atrial fibrillation (AF), undergoing percutaneous coronary intervention with stent implantation (PCI), has become the subject of intense clinical research aimed at defining the optimal antithrombotic strategy in this complex clinical setting [1,2,3,4,5,6,7,8]. This scenario is particularly challenging because it involves the need to simultaneously prevent two distinct but potentially competing risks: systemic thromboembolism related to AF or other indications for OAC and coronary thrombotic events, including stent thrombosis (ST) and recurrent myocardial infarction (MI), following PCI [9]. On the one hand, OAC is indicated for the prevention of thromboembolic complications, both arterial and venous, depending on the underlying clinical condition, including AF, mechanical prosthetic heart valves, and venous thromboembolism (VTE) [9]. On the other hand, dual antiplatelet therapy (DAPT), typically consisting of aspirin combined with a P2Y12 receptor inhibitor (clopidogrel, prasugrel, or ticagrelor), remains the standard of care after PCI to prevent ST and major adverse cardiovascular events (MACEs) [10,11]. The coexistence of these two indications therefore creates a therapeutic dilemma, as combining anticoagulant and antiplatelet therapy significantly increases the risk of bleeding complications [9]. Early observational data suggested that the combination of OAC with DAPT, the so-called triple antithrombotic therapy (TAT)—initially based on vitamin K antagonists (VKAs) such as warfarin, aspirin, and clopidogrel—was associated with a reduction in ischemic and thromboembolic events compared with less intensive regimens [9]. However, this benefit was counterbalanced by a substantial and clinically relevant increase in both total and major bleeding events, which in turn was associated with higher morbidity and mortality [9]. These findings highlighted the need for a more balanced approach able to preserve ischemic protection while minimizing hemorrhagic risk. In this context, a series of randomized controlled trials were designed and conducted to identify the optimal antithrombotic regimen in patients requiring both OAC and antiplatelet therapy after PCI. The WOEST [12], PIONEER AF-PCI [13], RE-DUAL PCI [14], AUGUSTUS [15], and ENTRUST AF-PCI [16] trials represent the cornerstone evidence in this field. These studies compared conventional TAT based on warfarin, aspirin, and clopidogrel with double antithrombotic therapy (DAT), consisting of OAC plus a single P2Y12 inhibitor, predominantly clopidogrel. Importantly, these trials also introduced and evaluated direct oral anticoagulants (DOACs), including rivaroxaban, dabigatran, apixaban, and edoxaban, replacing warfarin in the experimental arms of PIONEER AF-PCI [13], RE-DUAL PCI [14], AUGUSTUS [15], and ENTRUST AF-PCI [16], respectively (Table 1). Of note, the AUGUSTUS trial [15] uniquely employed a factorial design, allowing for the independent comparison of apixaban versus warfarin and aspirin versus placebo, and demonstrated a significant reduction in bleeding with apixaban-based strategies. Despite heterogeneity in study design, OAC dosing strategies, patient populations, and duration of antiplatelet therapy across trials, this studies consistently showed that DAT is associated with a similar efficacy in preventing ischemic events compared with TAT, but with a significantly improved safety profile, particularly in terms of major bleeding [12,13,14,15,16] (Table 1, Figure 1). However, a consistent numerical trend toward an early increase in ischemic events, particularly MI and ST, has been observed in patients treated with DAT compared with TAT, especially during the early post-PCI period when thrombotic risk is highest [17,18,19] (Table 1, Figure 1).

Based on this consistent body of evidence, current international guidelines recommend that in patients with AF, and by extension in those with other indications for OAC, undergoing PCI, a short course of TAT consisting of OAC, aspirin, and clopidogrel should be administered [20,21]. This initial phase is generally limited to up to 1 week, with the aim of covering the highest period of acute thrombotic risk immediately following PCI, when the risk of ST and peri-procedural MI is maximal [20,21]. Thereafter, a step-down strategy is recommended, transitioning to DAT with OAC plus a single P2Y12 inhibitor—most commonly clopidogrel—for a period of 6–12 months, depending on whether PCI was performed in a chronic or acute setting, respectively [20,21]. After completion of this phase, long-term management consists of OAC monotherapy, which has been consistently shown to be adequate also for the prevention of recurrent coronary thrombotic events [20,21,22,23]. Across all these treatment phases, and in the absence of contraindications, DOACs are generally preferred over VKAs, given their more favorable bleeding profile and more predictable pharmacokinetics [20,21]. As highlighted above, the preferential combination of clopidogrel with a DOAC in DAT is largely derived from the design of the pivotal randomized controlled trials, in which aspirin was systematically discontinued after the initial TAT phase, leaving clopidogrel as the sole antiplatelet agent [12,13,14,15,16]. While this strategy has been consistently associated with a reduction in bleeding events without loss of overall ischemic efficacy, it is important to recognize that it also reflects a pragmatic trial-driven simplification rather than a direct mechanistic comparison of different antiplatelet agents within DAT. This point is particularly relevant in light of the well-documented interindividual variability in response to clopidogrel, with high on-treatment platelet reactivity reported in up to 30% of patients [24,25]. In addition, pharmacokinetic interactions may further attenuate its antiplatelet effect. In particular, concomitant use of certain commonly prescribed drugs in patients with coronary artery disease undergoing PCI, such as atorvastatin, has been reported to potentially interfere with clopidogrel metabolism via CYP450 pathways [26]. Collectively, these factors raise the theoretical concern that in a subset of patients, early transition from TAT to OAC plus clopidogrel may result in suboptimal platelet inhibition during a period of persistent coronary vulnerability. On the other hand, clopidogrel has long been considered the P2Y12 inhibitor of choice in combination with OAC, largely because of its more favorable bleeding profile compared with more potent agents such as prasugrel or ticagrelor, which were underrepresented in the pivotal trials [12,13,14,15,16]. Moreover, comparative data from antiplatelet therapy in PCI populations without indications for OAC suggest superior efficacy of clopidogrel over aspirin in preventing coronary events [27]. This has led to the prevailing rationale that, when one antiplatelet agent must be withdrawn in patients requiring concomitant anticoagulation, clopidogrel represents the most appropriate compromise between ischemic protection and bleeding risk in the early post-PCI period [20,21].

## 2. Clopidogrel vs. Aspirin for Secondary Prevention

The first evidence suggesting superior efficacy of clopidogrel compared with aspirin in the context of atherothrombotic disease dates back to the CAPRIE trial [28]. In this large randomized study, 19,185 patients with established atherosclerotic vascular disease—manifesting as recent ischemic stroke, recent MI, or symptomatic peripheral arterial disease—were assigned to clopidogrel 75 mg once daily or aspirin 325 mg once daily and followed for a mean of 1.91 years [28]. Clopidogrel was associated with a statistically significant, relative risk reduction of 8.7% in the composite endpoint of ischemic stroke, MI, or vascular death (95% confidence interval [CI] 0.3–16.5; *p* = 0.043) [28]. Importantly, no significant differences in major bleeding were observed between the two treatment arms, suggesting a broadly comparable safety profile in this heterogeneous population [28]. Although the absolute treatment effect was limited, CAPRIE [28] established the biological and clinical rationale that P2Y12 inhibition with clopidogrel may provide at least comparable—and in selected settings superior—protection against atherothrombotic events compared with aspirin. More recently, this hypothesis has been revisited in the setting of patients undergoing PCI. In the HOST-EXAM trial [29], 5530 patients who had completed 6 to 18 months of uneventful DAPT after drug-eluting stent implantation were randomized to clopidogrel 75 mg once daily versus aspirin 100 mg once daily across 37 centers in South Korea. Over 24 months of follow-up, clopidogrel significantly reduced the composite endpoint of all-cause death, non-fatal MI, stroke, readmission due to acute coronary syndrome, and Bleeding Academic Research Consortium (BARC) type ≥ 3 bleeding (hazard ratio [HR] 0.73; 95% CI 0.59–0.90; *p* = 0.0035) [29]. This benefit was driven by both a reduction in ischemic events and clinically relevant bleeding, including any bleeding (HR 0.70; 95% CI 0.51–0.98; *p* = 0.036) and major bleeding (BARC ≥ 3: HR 0.63; 95% CI 0.41–0.97; *p* = 0.035) [29]. Of note, an early signal of increased all-cause and non-cardiovascular mortality observed in the clopidogrel arm [29] was not confirmed in the subsequent HOST-EXAM Extended follow-up analysis, which provided a median follow-up of 5.8 years (interquartile range 4.8–6.2) [30]. In this extended observation, the superiority of clopidogrel over aspirin was maintained, with a persistent reduction in the composite ischemic and bleeding endpoint (HR 0.74; 95% CI 0.63–0.86; *p* < 0.001), as well as in any bleeding (HR 0.74; 95% CI 0.57–0.94; *p* = 0.016) and BARC ≥ 3 bleeding (HR 0.65; 95% CI 0.47–0.90; *p* = 0.008) [30]. These findings strengthen the concept that long-term P2Y12 inhibition with clopidogrel may be at least as safe—and potentially safer—than aspirin in stable post-PCI patients. Further supporting this paradigm, the SMART-CHOICE trial [31] evaluated 5506 high-risk patients undergoing PCI (including those with prior MI, diabetes mellitus, or complex coronary anatomy) who had completed standard-duration DAPT. Patients were randomized at 26 sites in South Korea to receive either clopidogrel 75 mg once daily or aspirin 100 mg once daily after DAPT cessation [31]. At a median follow-up of 2.3 years (interquartile range 1.6–3.0), clopidogrel significantly reduced the risk of the composite endpoint of all-cause death, MI, or stroke compared with aspirin (HR 0.71; 95% CI 0.54–0.93; *p* = 0.013) [31]. In this study, the risk of bleeding was similar between groups (HR 0.97; 95% CI 0.67–1.42), suggesting that the observed benefit was primarily driven by a reduction in ischemic events rather than safety differences [31].

## 3. Clopidogrel vs. Aspirin for Secondary Prevention in OAC Patients

Building upon the evidence derived from dedicated randomized controlled trials [12,13,14,15,16] and the consequent guideline recommendations [20,21], in which clopidogrel was systematically selected as the preferred P2Y12 inhibitor to be continued after completion of the initial phase of TAT, it remained until recently unclear whether aspirin might be used instead of clopidogrel in DAT combination in patients receiving OAC after PCI. In this context, relevant complementary evidence has emerged from real-world data. An analysis of the Korea National Health Insurance Service database between 2013 and 2020 identified 9157 patients with AF who underwent PCI and were subsequently treated with DAT consisting of a DOAC plus a single antiplatelet agent, either clopidogrel or aspirin [32] (Table 2). To reduce treatment selection bias inherent to observational designs, patients were classified into clopidogrel or aspirin groups and subsequently matched using a 1:1 propensity score approach, resulting in 2882 well-balanced pairs [32]. During a median follow-up of 20.1 months (interquartile range 9.5–33.2), no statistically significant differences were observed between clopidogrel and aspirin in the incidence of MACEs, defined as a composite of cardiovascular death, MI, ischemic stroke, or systemic thromboembolism [32] (Table 2). Similarly, the risk of major bleeding events did not differ significantly between the two treatment strategies [32]. Importantly, no differences were also observed in net adverse clinical events (NACEs), a composite endpoint integrating both ischemic and bleeding outcomes, including all-cause death, MI, stroke, systemic embolism, or major bleeding [32]. Further insights into the comparative effectiveness of clopidogrel versus aspirin in the setting of DAT among OAC patients undergoing PCI have been provided by a prespecified subanalyses of the STOPDAPT-3 randomized clinical trial conducted in Japan, which compared a strategy of 1-month DAPT followed by aspirin monotherapy versus 1-month prasugrel monotherapy followed by clopidogrel monotherapy in patients with acute coronary syndromes or high bleeding risk undergoing PCI [33]. Among the 5809 patients enrolled in the STOPDAPT-3 trial [33], 788 (13.6%) were on OAC at discharge [34] (Table 2). In this prespecified subgroup, the incidence of both ischemic outcomes (a composite of cardiovascular death, MI, definite ST, or ischemic stroke) and bleeding events (BARC type 3 or 5) was comparable between the aspirin-based and clopidogrel-based strategies [34]. Although limited by the subgroup nature of the analysis, these findings suggest that the relative neutrality between aspirin and P2Y12 inhibition observed in the overall trial population may also extend to patients receiving concomitant OAC [33,34]. Additional evidence comes from the real-world, prospective, multicenter PERSEO registry in which 1234 patients with AF undergoing PCI were enrolled at 25 Italian centers [35]. Of the 989 patients who initiated DAT following an initial period of TAT, 769 (78%) were treated with clopidogrel-based DAT, whereas 220 (22%) received aspirin-based DAT [36] (Table 2). At a median follow-up of 12.3 months, the incidence of net adverse cardiac events (NACEs)—defined as a composite of major adverse cardiovascular and cerebrovascular events (including all-cause death, cardiac death, non-fatal MI, ST, non-fatal stroke or transient ischemic attack, and target vessel revascularization) and major or clinically relevant bleeding—was similar between the two treatment strategies [36]. Multivariable adjustment confirmed the absence of a significant association between type of DAT and clinical outcomes, and time-to-event analyses consistently demonstrated overlapping event curves for clopidogrel-based and aspirin-based DAT [36].

## 4. Discussion

**Figure 2:**
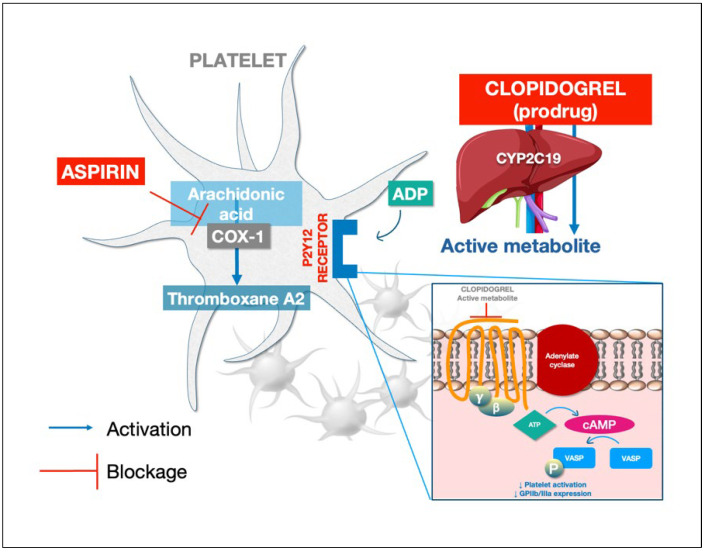
Mechanisms of action of aspirin and clopidogrel on platelet inhibition. Aspirin irreversibly inhibits cyclooxygenase-1, blocking the synthesis of thromboxane A_2_. Clopidogrel (prodrug) is converted in the liver to an active metabolite that irreversibly binds to the P2Y_12_ receptor, inhibiting the ADP-mediated signaling pathway, thus reducing GPIIb/IIIa expression and platelet aggregation. VASP: Vasodilator-Stimulated Phosphoprotein.

Overall, the available evidence summarized above suggests that in patients on OAC undergoing PCI, aspirin might be an alternative option to clopidogrel for DAT. Instead, in the setting of single antiplatelet therapy for secondary prevention of ischemic heart disease outside the context of long-term OAC, a growing body of evidence suggests that clopidogrel may provide superior ischemic protection compared with aspirin [29,30,31,37]. This apparent discrepancy raises the hypothesis that the presence of concomitant OAC may modulate or attenuate the relative pharmacodynamic differences between the two antiplatelet agents. To better interpret these clinical observations, the pharmacological mechanisms of aspirin and clopidogrel must be considered [38]. Although both agents ultimately reduce platelet activation and thrombus formation, they act through distinct and complementary biological pathways [38] (Figure 2). Aspirin exerts its antithrombotic effect through irreversible inhibition of platelet cyclooxygenase-1 (COX-1), thereby suppressing the synthesis of thromboxane A2, a potent mediator of platelet aggregation and vasoconstriction [38] (Figure 2). In contrast, clopidogrel irreversibly inhibits the platelet P2Y12 receptor, thereby blocking ADP-mediated platelet activation and amplifying downstream signaling pathways involved in platelet degranulation and aggregation [38] (Figure 2). Importantly, P2Y12 receptor signaling represents a central amplification pathway in platelet activation, integrating multiple upstream stimuli, including thromboxane A2, thrombin, collagen, and von Willebrand factor [38]. As a consequence, its inhibition may result in a broader suppression of platelet reactivity compared with the more selective upstream blockade of thromboxane A2 synthesis achieved with aspirin [38]. This mechanistic hierarchy provides a biological rationale supporting clinical observations from multiple randomized trials and comparative studies suggesting a potential advantage of clopidogrel over aspirin in reducing ischemic events in selected settings [29,30,31,37]. In the context of ongoing OAC however, the differential effect may be different. By reducing thrombin generation and activity—thrombin being the most potent platelet activator—OAC acts upstream of both aspirin and P2Y12-mediated pathways [38]. This upstream inhibition may therefore attenuate baseline platelet activation to a degree that reduces the relative incremental differences between aspirin- and clopidogrel-mediated platelet inhibition in patients treated with DAT. In this framework, the lack of consistent clinical superiority of either agent in patients receiving OAC after PCI [32,34,36] may reflect a “ceiling effect” of antithrombotic inhibition, whereby thrombin suppression partially neutralizes pathway-specific differences in platelet reactivity. A similar mechanistic consideration may also help explain why differences in bleeding outcomes between aspirin and clopidogrel appear minimal in the setting of concomitant OAC [32,34,36].

Moreover, it should be emphasized that the evidence supporting the superiority of clopidogrel over aspirin in contemporary antiplatelet strategies is not entirely consistent across randomized clinical trials [29,30,31,37]. In the HOST-EXAM [29,30] and SMART-CHOICE 3 [31] trials, where no patients with concomitant OAC were included, different patterns of benefit were observed across individual endpoints. Specifically, MI was significantly reduced with clopidogrel only in SMART-CHOICE 3 [31], whereas reductions in bleeding events and stroke were predominantly observed in HOST-EXAM [29,30]. Importantly, neither trial demonstrated a statistically significant difference in cardiac death or ST [29,30,31]. These discrepancies suggest that the observed benefit of clopidogrel may be driven primarily by composite endpoint effects rather than uniform superiority across all individual ischemic outcomes [29,30,31,37]. It is also important to note that both trials were conducted in East Asian populations, in which the so-called “East Asian paradox”—characterized by a relatively higher bleeding risk and a comparatively attenuated ischemic benefit from intensified antithrombotic therapy—has been consistently described [39]. This ethnic and clinical background may therefore limit the generalizability of the observed treatment effects to Western populations, where ischemic and bleeding risk profiles may differ [39]. In line with these considerations, a recent meta-analysis including seven studies and a total of 20,360 patients reported that clopidogrel was associated with superior efficacy for the composite endpoint of cardiac death, MI, and stroke compared with aspirin, while no significant differences were observed for individual ischemic endpoints [37]. These findings further support the hypothesis of a modest but consistent advantage of clopidogrel in composite ischemic outcomes, although without clear superiority in specific hard endpoints. Accordingly, the most recent ESC guidelines for the management of chronic coronary syndromes position clopidogrel and aspirin at a comparable level in patients with previous PCI, recommending clopidogrel as an alternative rather than a preferred agent for long-term antiplatelet therapy [11].

From a pharmacological standpoint, clopidogrel is also characterized by two clinically relevant considerations that may influence its effectiveness in real-world practice [25,40,41,42,43]. First, its antiplatelet activity may be affected by interindividual variability in drug response, largely driven by CYP2C19 genetic polymorphisms, which can result in reduced formation of its active metabolite and high on-treatment platelet reactivity in a subset of patients [25]. Second, a potential drug–drug interaction with proton pump inhibitors (PPIs) has been described, particularly with agents such as omeprazole and esomeprazole, which may inhibit CYP2C19-mediated activation of clopidogrel [40,41,42,43]. In patients receiving concomitant OAC and antiplatelet therapy, the use of PPIs is generally recommended to reduce the risk of gastrointestinal bleeding [11,12]. However, a recent subanalysis of the STOPDAPT-3 trial suggested that concomitant PPI use may be associated with a reduction in the antithrombotic efficacy of P2Y12 inhibition in terms of ischemic event prevention [40]. It should be noted that this interaction is not uniform across all PPIs, as clinically relevant differences in CYP2C19 inhibition exist among agents; omeprazole, esomeprazole, and lansoprazole appear to exert a greater inhibitory effect compared with pantoprazole and rabeprazole [40,41,42,43]. Taken together, these considerations highlight the importance of individualized therapeutic decision-making in patients requiring combined antithrombotic therapy. While clopidogrel remains a widely adopted and guideline-endorsed option, its pharmacogenetic variability and potential drug–drug interactions should be carefully considered when tailoring antithrombotic strategies, particularly in patients at high bleeding risk or requiring long-term PPI co-therapy.

Currently, no dedicated prospective studies have specifically evaluated the impact of CYP2C19 genotype–guided antiplatelet therapy in patients receiving DAT with OAC and clopidogrel after PCI [25]. While pharmacogenetic analyses have been extensively explored in the setting of DAPT, particularly in patients treated with P2Y12 inhibitors in combination with aspirin [25,44,45], direct evidence in the context of concomitant OAC remains lacking. In this specific clinical setting, reduced responsiveness to clopidogrel due to loss-of-function CYP2C19 alleles may theoretically have greater clinical relevance compared with standard DAPT populations. This is because, within DAT, clopidogrel represents the sole antiplatelet agent, and therefore its pharmacodynamic variability is not counterbalanced by a second antiplatelet drug such as aspirin. Consequently, any reduction in clopidogrel activation may translate into a more pronounced attenuation of platelet inhibition and potentially higher residual thrombotic risk during the early post-PCI period.

## 5. Conclusions

Based on the currently limited available evidence comparing the efficacy and safety of clopidogrel versus aspirin when used as the single antiplatelet agent within DAT in patients receiving OAC, most commonly for AF, undergoing PCI, no definitive superiority of either agent is apparent. Within this context, aspirin may represent a potential alternative to the more widely adopted strategy of clopidogrel-based DAT, although robust comparative data remain limited [32,34,36]. At present, the choice between clopidogrel and aspirin in this setting should possibly be individualized and left to the clinical judgement of the treating physician, taking into account the overall balance between ischemic and bleeding risk, as well as the relative strengths of the available evidence supporting each strategy. In addition to clinical risk stratification, several patient-specific factors may influence the effectiveness of clopidogrel, including interindividual variability in platelet response due to CYP2C19 genetic polymorphisms, as well as potential drug–drug interactions with concomitant medications such as certain proton pump inhibitors [25,40,41,42,43,44,45]. These considerations may be particularly relevant in patients in whom suboptimal platelet inhibition is suspected or in whom pharmacological interactions are unavoidable.

## 6. Future Directions

Large-scale randomized data directly comparing aspirin and clopidogrel when used within DAT in patients receiving OAC who have undergone PCI, particularly but not exclusively in the setting of AF, are needed to further strengthen the current data and to more definitively clarify the optimal choice of the single antiplatelet agent. At present, available evidence remains largely indirect and derived from subgroup analyses, observational studies, or extrapolation from trials not specifically designed to address this comparison [32,34,36]. In addition, the potential role of more potent P2Y12 inhibitors, such as prasugrel and ticagrelor, in combination with OAC warrants further systematic investigation. Although these agents are generally associated with greater platelet inhibition compared with clopidogrel, their safety and net clinical benefit in the context of concomitant OAC remain insufficiently defined. Limited data suggest that ticagrelor may have comparable efficacy and safety to clopidogrel when used in combination with dabigatran [46], but a generalized recommendation cannot be currently given. Finally, future research should also address whether individualized strategies based on platelet function testing and/or CYP2C19 genotype assessment may help guide the selection of the most appropriate antiplatelet agent—particularly P2Y12 inhibitors—in patients requiring concomitant OAC.

## Figures and Tables

**Table 1:** Study investigating triple versus double antithrombotic therapy after PCI.

RCT	WOEST	PIONEER AF-PCI	RE-DUAL PCI	AUGUSTUS	ENTRUST AF-PCI
OAC	VKA	Rivaroxaban	Dabigatran	Apixaban	Edoxaban
Inclusion criteria	OAC + PCI	AF + PCI (including stent implantation)	AF + PCI	AF + ACS and/or PCI	AF + PCI (including stent implantation)
Duration	12 months	12 months	Minimum 6 months	6 months	12 months
DUAL arm	VKA + Clopidogrel	Rivaroxaban 15 mg OD + P2Y12i	Dabigatran 150 mg BID + P2Y12i Dabigatran 110 mg BID + P2Y12i	Apixaban 5 mg BID + P2Y12i VKA + P2Y12i	Edoxaban 60 mg OD + P2Y12i
TRIPLE arm	VKA + Clopidogrel + Aspirin	Rivaroxaban 2.5 mg BID + DAPT VKA + DAPT	VKA + DAPT	Apixaban 5 mg BID + DAPT VKA + DAPT	VKA + DAPT
Primary endpoint	Any bleeding	TIMI clinically significant bleeding or bleeding requiring medical attention	Time to first ISTH major or CRNM bleeding event	Time to first ISTH major or CRNM bleeding at 6 months	Time to first ISTH major or CRNM bleeding event

Abbreviations. OAC, oral anticoagulant; VKA, vitamin K antagonist; PCI, percutaneous coronary intervention; AF, atrial fibrillation; ACS, acute coronary syndrome; OD once a day; BID, bis in die; DAPT, dual antiplatelet therapy; TIMI, thrombolysis in myocardial infarction; ISTH, International Society on Thrombosis and Haemostasis; CRNM, clinically relevant non-major.

**Table 2:** Studies investigating difference between an aspirin-based vs clopidogrel-based DAT on OAC patients undergoing PCI.

Reference	Population	Study Design	Endpoints	Follow-Up Time	Main Results
Kim et al. [32]	- 2882 patients receiving aspirin-based DAT * - 2882 patients receiving clopidogrel-based DAT * * from 9157 AF patients on DOAC undergoing PCI	Retrospective, nationwide, observational cohort study from the KNHIS	Primary - MACE (composite of CV death, MI, stroke, systemic TE) Secondary - All-cause death - Major bleeding - NACE (composite of all-cause death, MI, stroke, systemic embolism, major bleeding)	20.1 months	After PS matching, not significant difference in MACEs No difference in the incidence of ischemic endpoints, major bleeding or NACE
Natsuaki et al. [34]	- 405 patients on aspirin-based DAT * - 383 patients on clopidogrel-based DAT * * by the 30-day landmark analysis from the STOPDAPT-3 trial	Subgroup analysis from the prospective, multicenter, open-label, blinded STOPDAPT-3 RCT	Coprimary - Cardiovascular (composite of CV death, MI, definite ST, or ischemic stroke) - Bleeding (BARC 3 or 5)	12 months	- No significant interaction between OAC and the effect of aspirin versus clopidogrel on the coprimary endpoints - No significant difference in the incidence of the coprimary endpoints
Rubboli et al. [36]	- 769 patients on clopidogrel-based DAT * - 220 patients on aspiring-based DAT * * Selected after an initial TAT post-PCI	Subgroup analysis from the prospective, multicenter, Italian PERSEO registry	Primary - NACE (MACCE + major and clinically relevant bleeding) Secondary - MACCE - All-cause death - CV death - Non-fatal MI - ST - Non-fatal stroke/TIA - TVR - Major and clinically relevant bleeding	12.3 months	- Similar NACE incidence - Similar incidence of secondary endpoints - No association between type of DAT and outcomes at multivariable analysis

Abbreviations. AF, atrial fibrillation; DOAC, oral anticoagulant; OAC, oral anticoagulant; DAT, double antithrombotic therapy; KNHIS, Korean National Health Insurance Service; MACEs, major adverse cardiovascular events; CV, cardiovascular; MI, myocardial infarction; TE, thromboembolism; PS, propensity score; NACEs, net adverse clinical events; RCT, randomized controlled trial; TAT, triple antithrombotic therapy; TIAs, transient ischemic attack; PCI, percutaneous coronary intervention.

## Data Availability

No new data were generated with this research.
